# Pro-Inflammatory Activated Kupffer Cells by Lipids Induce Hepatic NKT Cells Deficiency through Activation-Induced Cell Death

**DOI:** 10.1371/journal.pone.0081949

**Published:** 2013-12-03

**Authors:** Tongfang Tang, Yongheng Sui, Min Lian, Zhiping Li, Jing Hua

**Affiliations:** 1 Division of Gastroenterology and Hepatology, Ren Ji Hospital, Shanghai Jiao Tong University School of Medicine, Shanghai Institute of Digestive Disease, Shanghai ,P.R.China; 2 Department of Medicine, Johns Hopkins University, Baltimore, Maryland, United States of America; Bambino Gesu' Children Hospital, Italy

## Abstract

**Background:**

Dietary lipids play an important role in the progression of non-alcoholic fatty liver disease (NAFLD) through alternation of liver innate immune response.

**Aims:**

The present study was to investigate the effect of lipid on Kupffer cells phenotype and function *in* *vivo* and *in* *vitro*. And further to investigate the impact of lipid on ability of Kupffer cell lipid antigen presentation to activate NKT cells.

**Methods:**

Wild type male C57BL/6 mice were fed either normal or high-fat diet. Hepatic steatosis, Kupffer cell abundance, NKT cell number and cytokine gene expression were evaluated. Antigen presentation assay was performed with Kupffer cells treated with certain fatty acids *in* *vitro* and co-cultured with NKT cells.

**Results:**

High-fat diet induced hepatosteatosis, significantly increased Kupffer cells and decreased hepatic NKT cells. Lipid treatment *in* *vivo* or *in* *vitro* induced increase of pro-inflammatory cytokines gene expression and toll-like receptor 4 (TLR4) expression in Kupffer cells. Kupffer cells expressed high levels of CD1d on cell surface and only presented exogenous lipid antigen to activate NKT cells. Ability of Kupffer cells to present antigen and activate NKT cells was enhanced after lipid treatment. In addition, pro-inflammatory activated Kupffer cells by lipid treatment induced hepatic NKT cells activation-induced apoptosis and necrosis.

**Conclusion:**

High-fat diet increase Kupffer cells number and induce their pro-inflammatory status. Pro-inflammatory activated Kupfffer cells by lipid promote hepatic NKT cell over-activation and cell death, which lead to further hepatic NKT cell deficiency in the development of NAFLD.

## Introduction

The prevalence of non-alcoholic fatty liver disease (NAFLD) is increasing worldwide and is often linked with obesity and metabolic syndrome[1,2]. NAFLD ranges from simple steatosis (fatty liver) to non-alcoholic steatohepatitis (NASH), which can progress to cirrhosis and hepatocellular carcinoma. The pathogenesis of NAFLD is often interpreted by the ‘double-hit’ hypothesis. Recently, it has become apparent that NAFLD is metabolic disease characterized by insulin resistance and a low-grade inflammation, and growing evidence has demonstrated correlative and causative relationship between inflammation and insulin resistance[3,4]. More recently, increasing emphasis has been placed on altered innate immune response as a key event in the development of low-grade systemic chronic inflammation in such condition[5,6]. 

The liver contains enriched innate immune cells, such as macrophages (Kupffer cells), NK cells and natural killer T (NKT) cells[7]. Kupffer cells represent the largest group of fixed macrophages in the body and account for about 20-25% of non-parenchymal cells in the liver[8]. Kupffer cells are critical components of the innate immune system, they reside within the sinusoidal vascular space and can be activated by various endogenous and exogenous stimuli including lipopolysaccharide (LPS). Kupffer cell-derived cytokines, such as tumor necrosis factor-α (TNFα), play a key role in regulating the phenotype and function of neighbouring parenchymal and non-parenchymal cells[9]. In addition, Kupffer cells are potential antigen-presenting cells (APC) and participate in the liver T cell activation and tolerance. Consequently, modified Kupffer cells phenotype and function are essential in the development of various chronic and acute liver disease. In recent years, increasing evidence has shown the role of Kupffer cells in the pathgenesis of NAFLD[10,11]. Selective depletion of Kuppfer cells using gadolinium chloride (GdCl_3_) protects the mice against the development of diet-induced hepatic steatosis and insulin resistance[12].

NKT cells are a group of “unconventional” T cells that express both natural killer (NK) receptors and T cell receptors [13]. NKT cells specifically recognize glycolipid antigens, such as a synthetic lipid antigen α-galactosylceramide (αGalCer), which presented by the atypical major histocompatibility complex (MHC) class I-like molecule CD1d, and produce both Th1 (INF-γ )and Th2 (IL-4) cytokines when activated[14,15]. They are most abundant in liver and reside mainly in the hepatic sinusoids and balance the production of pro-inflammatory and anti-inflammatory cytokines[16]. Previous studies have shown that high fat diets fed mice or leptin-deficient ob/ob mice appeared increase of hepatic NKT cell apoptosis and NKT cell deficiency[17,18], which led to local and systematic inflammatory conditions that contributed to insulin resistance and fatty liver disease. Furthermore, such NKT cells alternation skewed other leukocytes toward proinflammatory cytokine production and promoted sensitization to LPS liver injury [17]. Restoring NKT cell deficiency by adoptive transfer in mice model of NAFLD reduces hepatic steatosis and insulin resistance[19]. Furthermore, our recent study have shown that hepatocytes mediated impaired CD1d-dependent endogenous antigen presentation due to dysfunction of lipid homeostasis may contribute to hepatic NKT cell depletion[20]. The results clearly showed the contribution of hepatocytes to the mechanism of high-fat diet induced heaptic NKT cell depletion. However, so far, few studies have been taken to investigate the direct interaction between Kupffer cells and NKT cells, both of them reside in the hepatic sinusoids and are important in the development of NAFLD. Importantly, the functional properties of NKT cells appeared to be modulated by professional APCs, such as dentritic cells[21]. 

In the current study, we first evulated the effect of high fat diet or fatty acids treatment on abundance and function of Kupffer cells. Furthermore, we investigate the impact of lipid treatment on ability of Kupffer cells antigen presentation to NKT cell and exploring the possible mechanism involved. 

## Materials and Methods

### Ethics statement

All animal experiments fulfilled Shanghai Jiao Tong University criteria for the humane treatment of laboratory animals and was approved by the Ren Ji Hospital Animal Care and Use Committee (SCXK2007-0005).

### Animal and treatments

Adult (age 6-8 week) male wild type C57BL/6 mice were obtained from Experimental Animal Center (Ren Ji Hospital, Shanghai Jiao Tong University). Mice were fed either a normal control diet (NC, 12% kcal from fat) or a high-fat diet (HF, 59% kcal from fat, Shanghai SLACCAS Company) for 12-16 weeks. All mice were maintained in a temperature- and light-controlled facility and permitted to consume water and pellet chow *ad libitum*. 

### Kupffer cells, hepatocytes and HMNCs Isolation

Kupffer cells were isolated by *in situ* perfusion of the liver as described previously[22]. Briefly, the liver was perfused through the portal vein with 100 mL of Ca^2+^- and Mg^2+^-free Hanks’ balanced salt solution (HBSS, Gibco, USA) at 37°C at a flow rate of 5 mL/min. Subsequent perfusion with complete HBSS containing 0.05% collagenase IV and 0.025% collagenase I (Sigma Aldrich, USA) at 37°C for 10 min. The liver was then excised and minced before incubation with HBSS/collagenase IV solution with continuous stirring at 37°C for 20 min. The liver slurry was filtered through 100-mesh gauze and washed with HBSS. Cells were then centrifuged using a discontinuous gradient of 25% percoll and 50% percoll (GE Healthcare, USA) at 800*g* for 20 min. Kupffer cell-enriched fraction was collected from the interface layer between the two gradients and washed with RPMI-1640 (Gibco, USA). Cells were seeded onto culture plates and nonadherent cells were removed after 2 h culture. Kupffer cells were recollected and plated onto a 24-well plate at a density of 5X10^5^/mL, and cultured in Dulbecco’s modified Eagle’s medium (DMEM, Gibco, USA) solution with 15% fetal bovine serum (FBS, Gibco, USA), supplemented with 100 U/mL of penicillin G, and 100 U/mL of streptomycin sulfate at 37°C with 5% CO_2_. 

Hepatocytes were isolated by *in situ* perfusion of the liver then cultured in DMEM solution on collagen I-coated plates as described previously[23]. Hepatic mononuclear cells (HMNCs) were isolated as described previously[20]. Cell viability was assessed by trypan blue. Exclusion was greater than 95% for Kupffer cells and greater than 90% for hepatocytes. 

### Cell surface labeling

After isolation, Kupffer cells were labeled with surface marker anti-mouse fluorescent antibody against CD1d and HMNCs were labeled with CD1d tetramers (NIH tetramer facility) loaded with a ligand (PBS-57, an analog of α-galactosylceramide (GalCer)) or anti-mouse fluorescent antibodies against NK1.1 and CD3 (Pharmingen, San Diego, USA). After surface labeling, Kupffer cells or HMNCs were evaluated by flow cytometry (Becton Dickinson, USA), and the data analyzed using FACSDiva version 6.2. For apoptosis and necrosis assay, cells in co-culture system were collected and stained with Annexin V and the vital dye 7-aminoactinomycin D (7-AAD), and simultaneously stained with CD3 and NK1.1, then evaluated by flow cytometry.

### In vitro fatty acids treatment of Kupffer cells

After isolation, Kupffer cells were plated onto a 24-well plate at a density of 5X 10^5^/mL in DMEM culture medium with 15% FBS. After 2h culture, supernatant was removed and fresh medium containing individual free fatty acids palmitoleic acid (PA) and/or oleatic acid (OA) (Sigma-Aldrich, OA/PA 0.5mmol/L) was administrated. After 24h incubation, Kupffer cells were collected and total RNA were extracted for mRNA expression analysis.

### Kupffer cells and hepatocytes antigen presentation to NKT cell line

Kupffer cells and hepatocytes were isolated as above and unloaded or loaded with αGalCer (100ng/ml, Enzo Biochemical, USA) in 96-well plate. After removing the media and washing extensively, V14^+^ mouse CD1d-specific NKT hybridoma cells (DN32.D3) were added into culture system and incubated with Kupffer cells or hepatocytes for 20h. IL-2 released from NKT cells to the media were determined by commercially available, mouse-compatible ELISAs (R&D systems,Inc, Minneapolis, USA).

### Effect of Fatty Acids or LPS Treatment on Lipid antigen presentation of Kupffer cellss

Kupffer cells were isolated and incabuted with palmitoleic acid (0.5mmol/L) or LPS (100ng/ml) overnight with or without loading αGalCer . The next day, HMNCs (served as hepatic NKT cells source) were isolated and added into culture system for co-culture 24h after removing the media and washing extensively. IFN-γ and IL-4 levels in culture supernatant were determined by mouse-compatible ELISA (R&D systems, USA). Cells were collected for RNA isolation.

### RNA Isolation and Evaluation of Cytokines Gene Expression

Quantization of the expression level of selected genes was performed by quantitative real-time polymerase chain reaction (PCR). Total RNA was obtained from liver tissues or Kupffer cells and cocultured cells with TRIzol reagent (Invitrogen, USA) and reverse-transcribed with Primescript RT Reagent kit (TaKaRa, Japan) according to manufacturer specifications. For real-time PCR, 10 ng of template was used in a 10-ul reaction containing each primer, SYBR Green PCR Master Mix (TaKaRa, Japan). All reactions were performed in triplicate using the following cycling conditions: 30 s at 95 °C, followed by 40 cycles of 95 °C for 5 s, 60 °C for 30 s and 72 °C for 30 s using ABI Prism 7300 system (Applied Biosystems, USA). The mean value of the triplicate for each sample was calculated and expressed as cycle threshold (CT). The amount of gene expression was then calculated as the difference (OCT) between the CT value of the sample for the target gene and the mean CT value of the endogenous control (β-actin). The relative level of expression was measured as 2-OOCT.

Mouse primers (provided by Shen Gong Biochemcial Co., Shanghai, China) were as follows

IFN-γ:TCAAGTGGCATAGATGTGGAAGAA and TGGCTCTGCAGGATTTTCATG.

TNF-α:CCAGGCGGTGCCTATGTCTC and CAGCCACTCCAGCTGCTCCT.

IL-10:GCTCTCGCAGCTCTAGGAGCATGTG and CGCAGCTCTAGGAGCATGTG.

TLR-4:GACACTTTATCCAGAGCCGTTG and GGACCTCTCCACTTTCTCAAGG.

β-actin:CTAAGGCCAACCGTGAAAAG and GGTACGACCAGAGGCATACA

### Liver Histology and Immunohistochemistry

For histological examination, liver tissue was fixed in 10% formalin, embedded in paraffin, sectioned, and stained with hematoxylin and eosin. For immunohistochemistry, the liver sections were pre-incubated with 5% bovine serum albumin for 10 min, and then incubated with anti-mouse F4/80 antibody (1:100 dilution, GeneTex Co., USA) for 12 hours at 4°C in a wet chamber. After washing with phosphate buffer saline (PBS, Gibco, USA), the sections were incubated with an anti-goat secondary antibody (Zhongshan Golden Bridge Biotech, Beijing, China) for 20 min at room temperature, and detected with diaminobenzidine (DAB) and hematoxylin as the counter stain. F4/80 positive cells were detected and semi-quantified on 10 fields of 200x magnification section using an Olympus light microscope. 

### Statistical analysis

All values are expressed as mean ± SEM. Treatment related differences were evaluated by one-way ANOVA. The paired-individual means were compared by t-test. Staistical significance was considered at *P*<0.05.

## Results

### High-fat diet increases Kupffer cells, reduces hepatic NKT cells and induces hepatosteatosis

**Figure 1:**
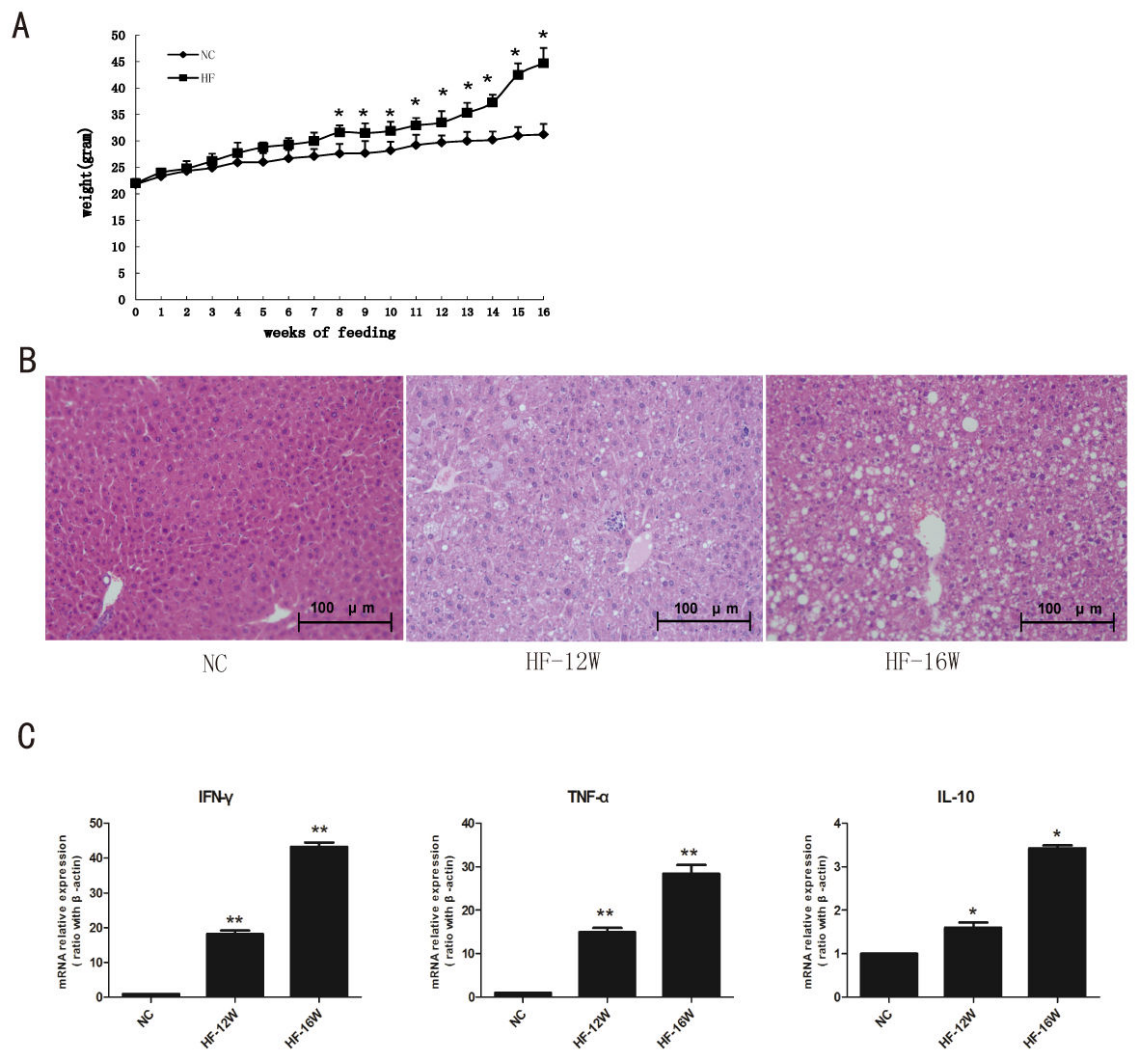
High-fat diet induce obesity, hepatic steatosis, and increased hepatic cytokine expression. Wild-type C57BL/6 mice were fed either normal diet or high-fat diet for 12 or 16 weeks.(A) Animal weight, (B) Liver histology (200x magnification), and (C) Hepatic cytokine expressions, determined by quantitative real time PCR. Values are mean ±SEM, * *P*<0.05,**P<0.01 versus NC; NC: normal diet control, HF:high fat diet; n=5 animals per group.

**Figure 2:**
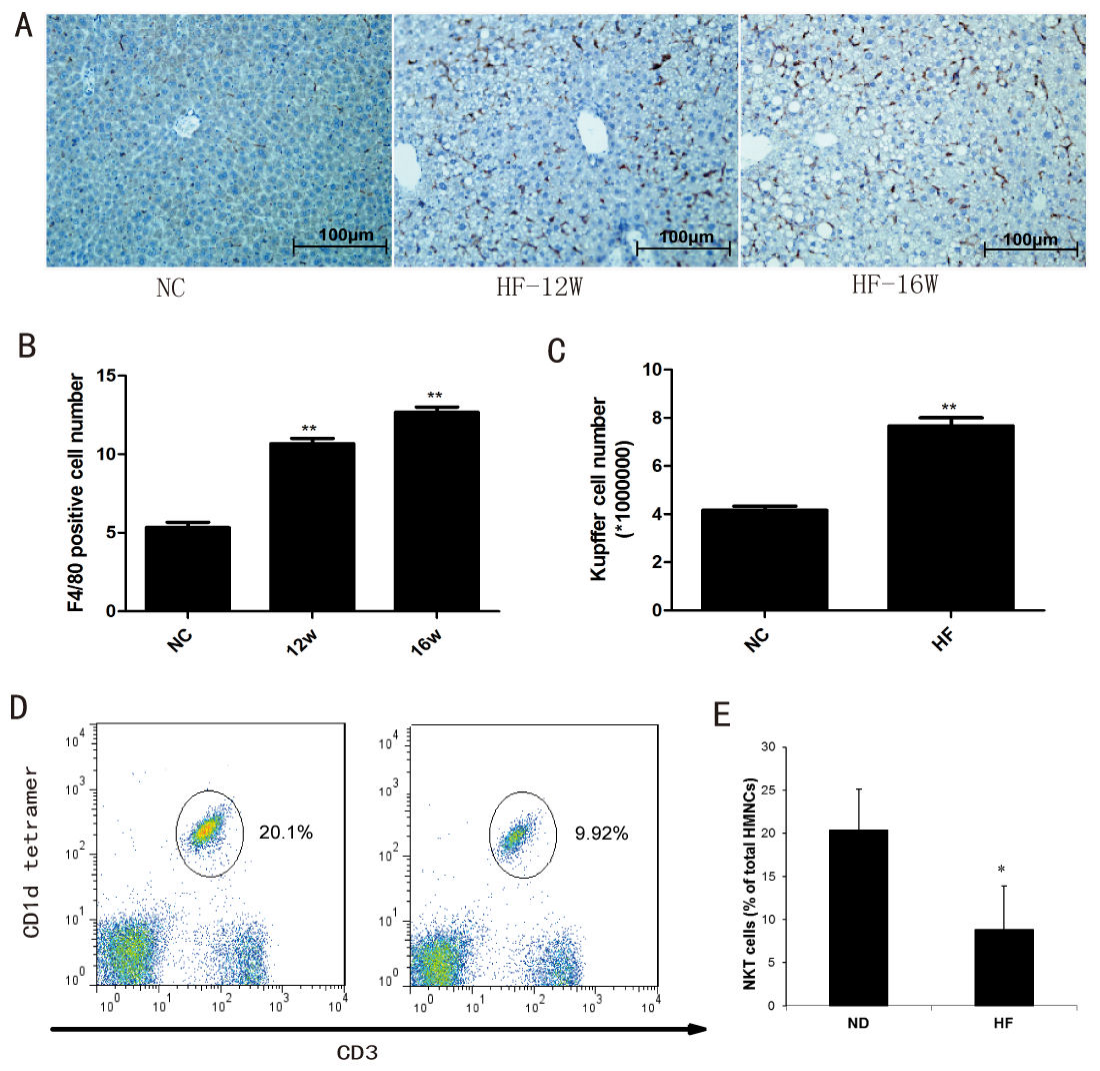
High-fat diet increase Kupffer cells and decrease NKT cells. Wild-type C57BL/6 mice were fed either normal diet or high-fat diet for 12 or 16 weeks. Kupffer cells were determined by immunohistochemistry staining or isolated through collagen perfusion. Hepatic mononuclear cells (HMNC) were isolated and NKT cells were identified with CD1d tetramer and CD3 double positive cells by flowcytometry. (A) F4/80 positive cells (Kupffer cells) distribution detected by immunohistochemistry (200x magnification), (B) Semi-quantitation of F4/80 positive cells, sections from mice fed either NC or HF were stained with F4/80.Ten fields per section from each mouse were analysed. (C) The numbers of isolated Kupffer cells form NC or HF feeding. (D) Representative dot plots of hepatic NKT cells (CD1d tetramer+CD3+) , (E) Mean (±SEM) results of hepatic NKT cell percentage. Values are mean ±SEM, * *P*<0.05,**P<0.01 versus NC, n=4 animals per group.

Increasing evidence have shown activation of Kupffer cells was an essential element in the pathogenesis of NAFLD. However, the number change of Kupffer cells in NAFLD still be controversial. In the current study, C57BL/6 mice were fed either high-fat diet or normal control diet for 12-16 weeks. As previously reported, high fat diet induced greater weight gain (Figure 1A), clear hepatosteatosis but no obvious inflammatory cells infiltration (Figure 1B). Simultaneously, the cytokines expression in steatotic liver tissues were significantly increased (Figure 1C). Kupffer cells were detected as F4/80 positive cells by immunohistochemical staining. As shown in Figure 2A, high-fat diet led to a significant increase of Kupffer cells. Semi-quantitive analysis showed almost two-fold increase of Kupffer cells aftering 16 weeks feeding in steatotic liver than that in normal liver (Figure 2B). In addition, Kupffer cells occurred morphology changes, presented with the marked enlargement in cell size (Figure 2A). To further confirmed the immunohistochemical results, Kupffer cells were isolated from high-fat diet or normal diet fed mice. The results demonstrated significant increase of Kupffer cells in high-fat diet fed mice than those in normal diet fed mice (Figure 2C). As our previous reported, high-fat diet reduced hepatic NKT cells as assessed by percentages of CD3 and CD1d tetramer double positive cells (Figure 2D,E). Together, these results indicated hepatostaetosis induced by high-fat diet was associated with changes in local cytokine patterns and altered hepatic innate immune system.

### High-fat diet and fatty acids treatment increase pro-inflammotory cytokines gene expression and TLR4 expression in Kupffer cells

**Figure 3:**
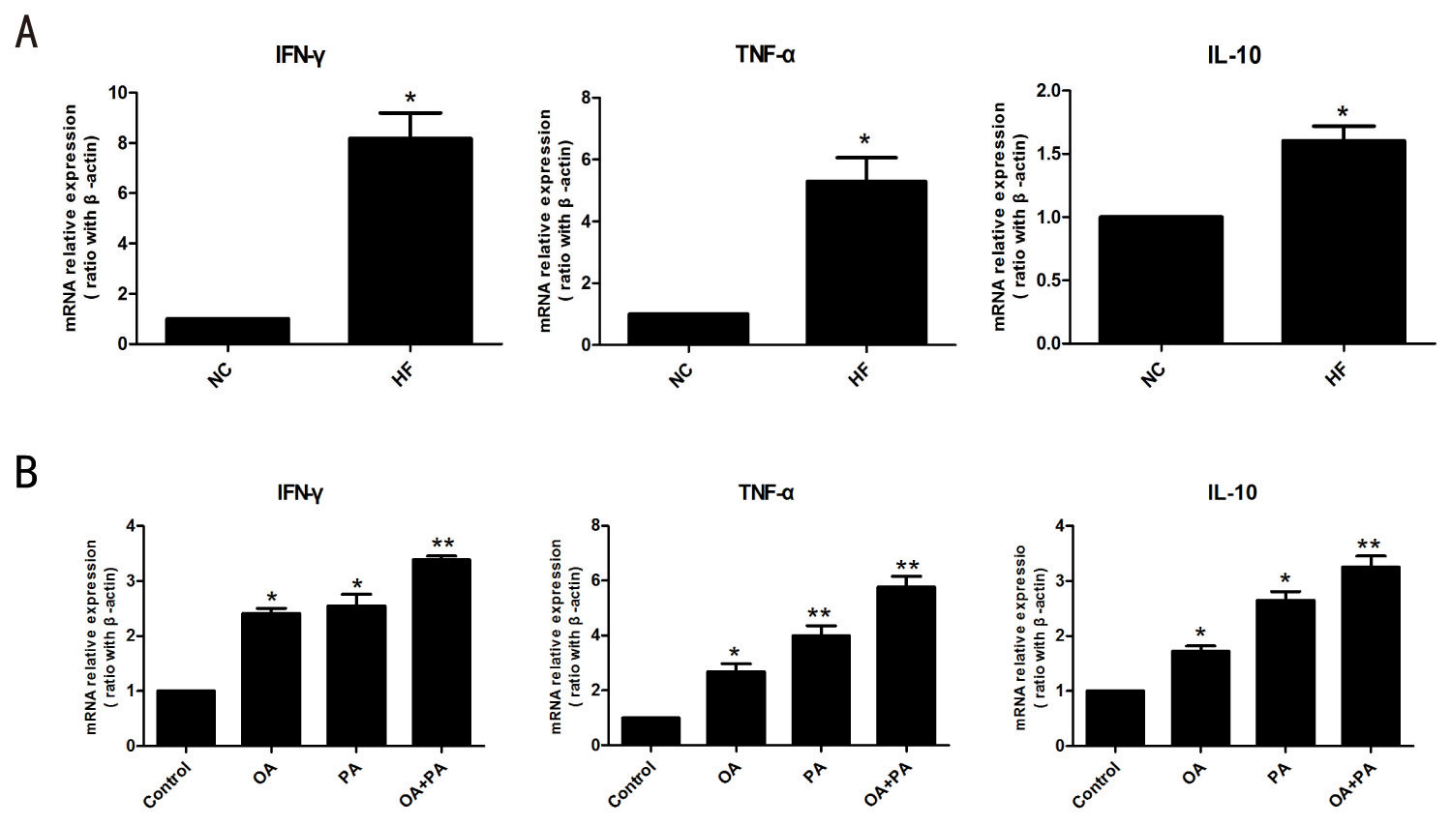
Cytokine gene expression in Kupffer cells after lipid treatment. (A) Cytokine gene expression on Kupffer cells after high-fat diet feeding. Wild-type C57BL/6 were fed either normal diet or high-fat diet for 16 weeks. Kupffer cells were isolated and cytokine gene expression were determined by quantitative real-time PCR. (n=4/group). (B) Cytokine expression on Kupffer cells after certain fatty acids treatment. Kupffer cells isolated from normal diet mice were treated with certain fatty acids (PA ,OA) for 24 h. (n=3/group). (PA,OA, 0.5mmol/L), Values are means±SEM, *P<0.05,**P<0.01 versus normal control.

**Figure 4:**
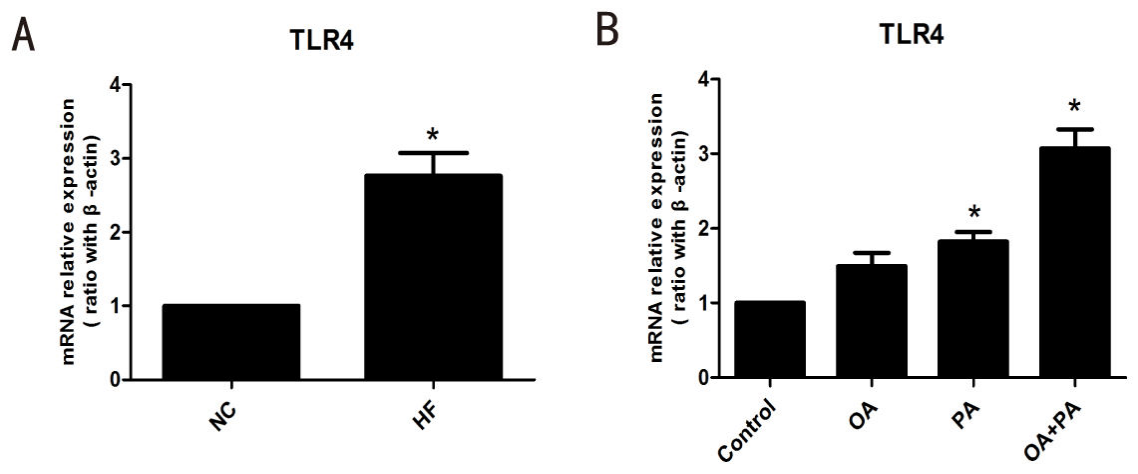
TLR4 expression in Kupffer cells after lipid treatment. (A) High-fat diet induced increased expression of TLR4 in Kupffer cells. (B) Fatty acids treatment induced increased expression of TLR4 in Kupffer cells. Values are means±SEM, *P<0.05 versus normal control, (n=4/group).

Previous studies have shown hepatosteatosis is associated with activation of hepatic inflammation singaling and shifting toward Th1 response[24]. To study the direct effect of lipid accumulation on Kupffer cells cytokine expression, Kupffer cells were isolated form high-fat diet fed mice or normal diet mice. Indeed, high-fat diet markedly increased expression of pro-inflammatory cytokine TNF-α and INF-γ in Kupffer cells, both of them are important in Kupffer cell-mediated liver injury (Figure 3A). Similar results were observed in Kupffer cells isolated from normal diet mice and treated *in vitro* with saturated fatty acids (PA) and monounsaturated fatty acids (OA), which have been showed to induce hepatosteatosis and insulin resistance in our previous study (Figure 3B)[20]. In addition, anti-inflammatory cytokine IL-10 gene expression was also increased in Kupffer cells after *in vivo or in vitro* lipid treatment. However, the increase extent of IL-10 was lower than those of TNF-α and INF-γ. Therefore, Th1-favored pro-inflammatory activation of Kupffer cells developed after *in vivo or in vitro* lipid treatment. Consistent with the potential activation of Kupffer cells was significant increase in toll-like receptor 4 (TLR4) expression in Kupffer cells after *in vivo* (high-fat diet, Figure 4A) or *in vitro* (fatty acids treatment, Figure 4B) lipid treatment. TLR4 signaling had been detected to involve fatty acids-induced activation of adipocytes and other macrophages[25,26]. Together, these results indicated that lipid treatment could directly induce pro-inflammatory activation of Kupffer cells through TLR4 signaling.

### Kupffer cells present exogenous but not endogenous lipid antigen to activate NKT cells

**Figure 5:**
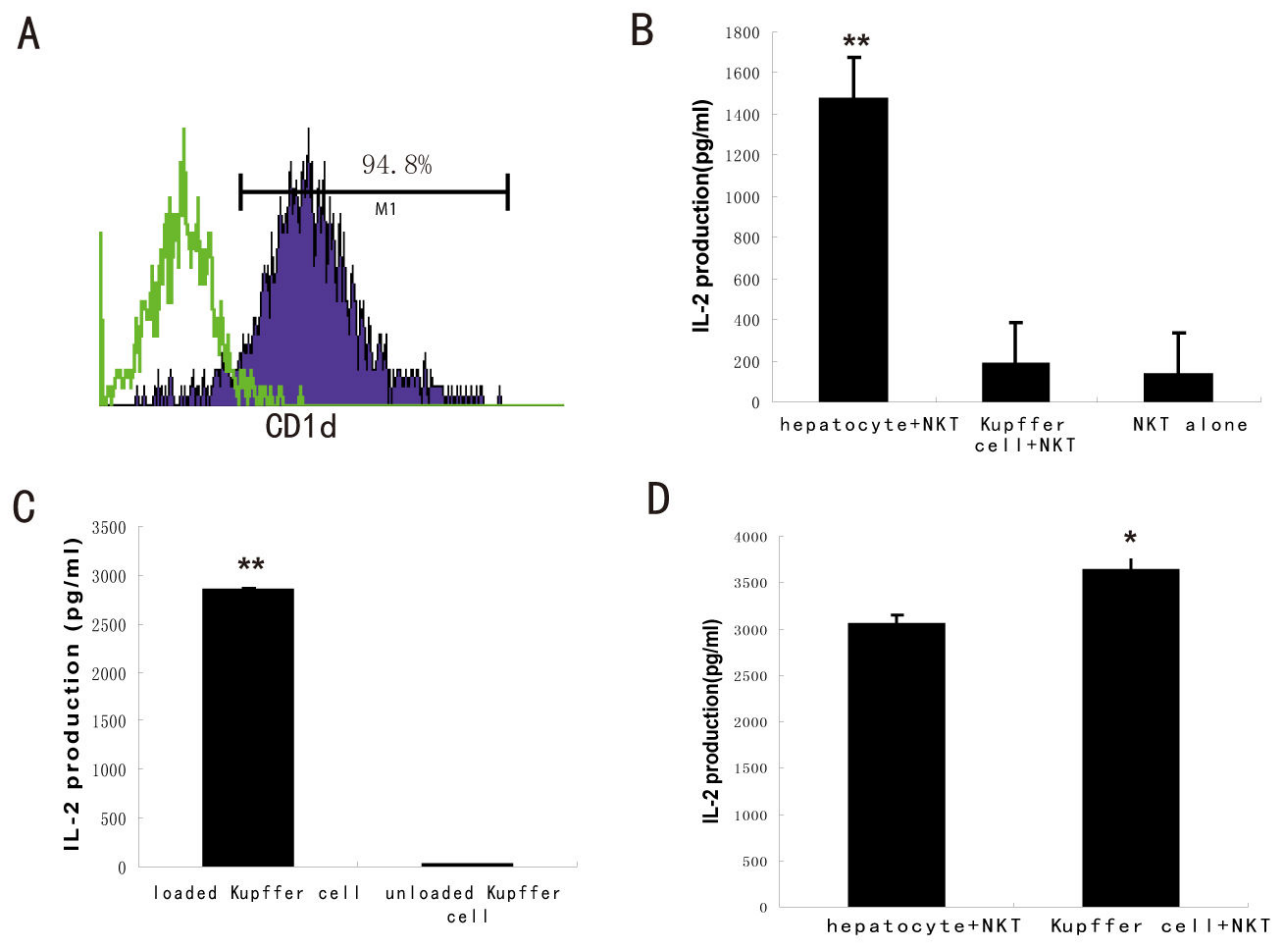
Kupffer cell present exogenous but not endogenous antigen to NKT cell. Kupffer cells and hepatocytes were isolated from wild-type mice. (A) Representative histogram of Kupffer cell CD1d expression. Green line indicates staining with isotype control. (B) Endogenous antigen presentation of hepatocytes and Kupffer cells. Kupffer cells and hepatocytes were isolated and co-cultured with a NKT cell line. IL-2 released from NKT cells indicated activation of NKT cells by hepatocytes or Kupffer cells. IL-2 levels in culture supernatant were determined by ELISA. (**P<0.01 versus NKT alone). (C) Exogenous antigen presentation of Kupffer cells. Kupffer cells were isolated and loaded with αGalCer (100ng/ml), then co-cultured with NKT cell line for 24h. IL-2 levels in culture supernatant was determined. (**P<0.01 versus unload Kupffer cell group). (D) Comparion of ability of exogenous lipid antigen presentation of hepatocytes and Kupffer cells. (*P<0.05 versus hepatocyte group) . Values are means±SEM, n=3/group.

Kupffer cells are potential antigen-presenting cells and participate in the liver T cell activation and tolerance. NKT cells recognize endogenous and exogenous lipid antigens presented by the MHC class I like molecule, CD1d. Normal Kupffer cells constitutely expressed high levels of CD1d on cell surface (94.8%, Figure 5A,). High fat diet or fatty acids treatment had no effect on CD1d expression on Kupffer cells. To investigate whether Kupffer cells can present endogenous lipid antigen through CD1d molecular like that of hepatocytes[20], Kupffer cells were incubated with the NKT cell hybridoma, DN32.D3, without loading exogenous lipid antigen. Unlike hepatocytes, Kupffer cells hardly activitated NKT hybridoma, as reflected by almost no production of IL-2 in culture supernature (Figure 5B). However, when Kupffer cells were loaded with an exogenous ligand, a synthetic ligand αGalCer, they showed strong ability to stimulate NKT hybridoma, as reflected by significantly increased IL-2 production (Figure 5C). Interestingly, the ability of Kupffer cells to present exogenous antigen and activate NKT cell was even stronger than that of hepatocytes (Figure 5D). These results indicate that Kupffer cells had no endogenous lipid antigen but can present exogenous lipid antigen to activate NKT cells.

### Fatty acids treatment enhanced Kupffer cells ability on antigen presentation and activation of NKT cells

**Figure 6:**
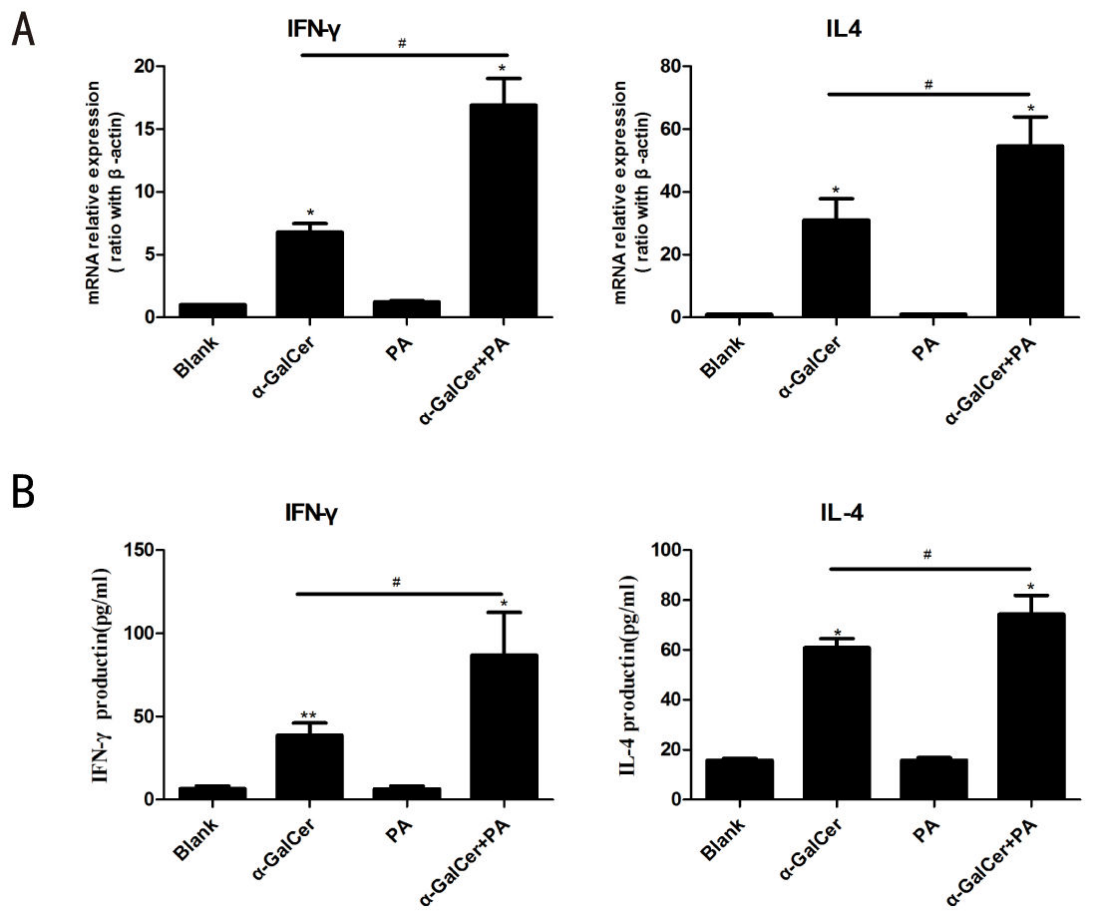
Fatty acids treatment increase Kupffer cells ability on antigen presentation and activation of NKT cells. Kupffer cells were isolated from wild-type C57BL/6 mice and treated with fatty acids (PA, 0.5mmol/L) and loaded with αGalCer (100ng/ml) overnight, and co-cultured with HMNC (served as NKT cells source) for 24h. Cells and culture supernatant were collected for detection of cytokine mRNA expression and protein levels. Increased expression of IFN-γand IL-4 indicated activation of NKT cells by αGalCer-loaded Kupffer cells. (A) IFN-γand IL-4 mRNA expression in co-culture system determined by real time PCR. (B) IFN-γand IL-4 levels in culture supernatant determined by ELISA. Values are means±SEM , *P<0.05, **P<0.01 versus Blank, ^#^ *P*<0.05 αGalCer group versus αGalCer+PA group, n=3 experiment.

As above shown, lipid treatment could induce pro-inflammatory activation of Kupffer cells, therefore, we’d like to further investigate whether pro-inflammatory activated Kupffer cells by lipids have impact on their antigen presentation and NKT cell activation. To more directly address this issue, a co-culture system composed of Kupffer cells and primary NKT cells (derived from HMNCs) was used. We measured IFN-γand IL-4 expression in co-culture system, which were expressed and secreted primarily by activated NKT cells when only stimuli was from αGalCer-loaded Kupffer cells. Antigen loaded Kupffer cells induced primary NKT cells activation as shown by significant mRNA expression of IFN-γand IL-4 in collected cells (Figure 6A), and high levels of IFN-γand IL-4 in co-culture media (Figure 6B). Furthermore, fatty acids treated Kupffer cells significantly increased ability to activate NKT cells, indicated by higher levels of IFN-γand IL-4 expression in fatty acids treatment group than those without (Figure 6A,B). However, fatty acids treatment alone had no effect on NKT cell activation. Therefore, pro-inflammatory activated Kupffer cells by lipid treatment may enhanced lipid antigen presentation and activation of NKT cells.

### LPS has a synergistic effect on Kupffer cells lipid antigen presentation and NKT cell activation

**Figure 7:**
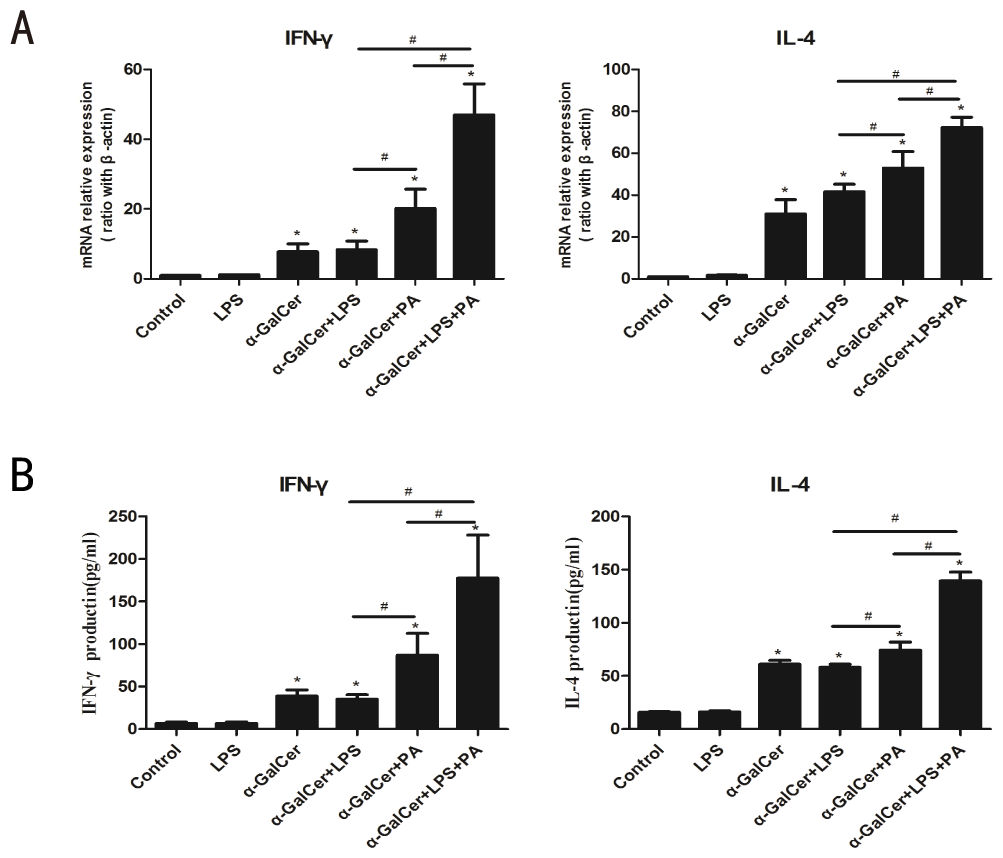
LPS had synergistic effect on Kupffer cells ability of activation of NKT cells. Kupffer cells were isolated from wild-type C57BL/6 mice and treated with fatty acids (PA 0.5mmol/L) and/or LPS (100ng/ml) in the presence of αGalCer (100ng/ml) overnight, then co-cultured with HMNC for 24h. (A) IFN-γand IL-4 mRNA expression in co-culture system. (B) IFN-γand IL-4 levels in culture supernatant determined by ELISA. Values are means±SEM , *P<0.05 versus control, ^#^ *P*<0.05 versus respective control , n=3 experiment.

LPS can stimulate activation of Kupffer cell through TLR4 receptor[27]. Activation of Kupffer cells by LPS played important roles in the mechanism of various acute or chronic liver disease. Furthermore, LPS levels was increased in human or animal models of NASH [28]. To further investigate whether LPS had synergistic effect on ability of Kupffer cells lipid antigen presentation, we isolated Kupffer cells and treated with LPS and/or fatty acids and co-cultured them with primary hepatic NKT cell. As shown in Figure 7A and 7B, LPS alone had no effect on NKT cell activation. After loading exogenous αGalCer, LPS treatment or fatty acids treatment enhanced Kupffer cells to present antigen and activate NKT cells as reflected by significant expression of IFN-γand IL-4 at the mRNA and protein levels in co-culture system (Figure 7A,B). When in the presence of both LPS and fatty acids, primary NKT cells activation can be further enhanced by Kupffer cells, indicated by highest expression of IFN-γand IL-4 at mRNA and protein levels. These results indicate LPS had a synergistic effect with fatty acids on Kupffer cells lipid antigen presentation and NKT cell activation.

### Pro-inflammatory activated Kupffer cells by lipid induce NKT cell activation-induced cell death

**Figure 8:**
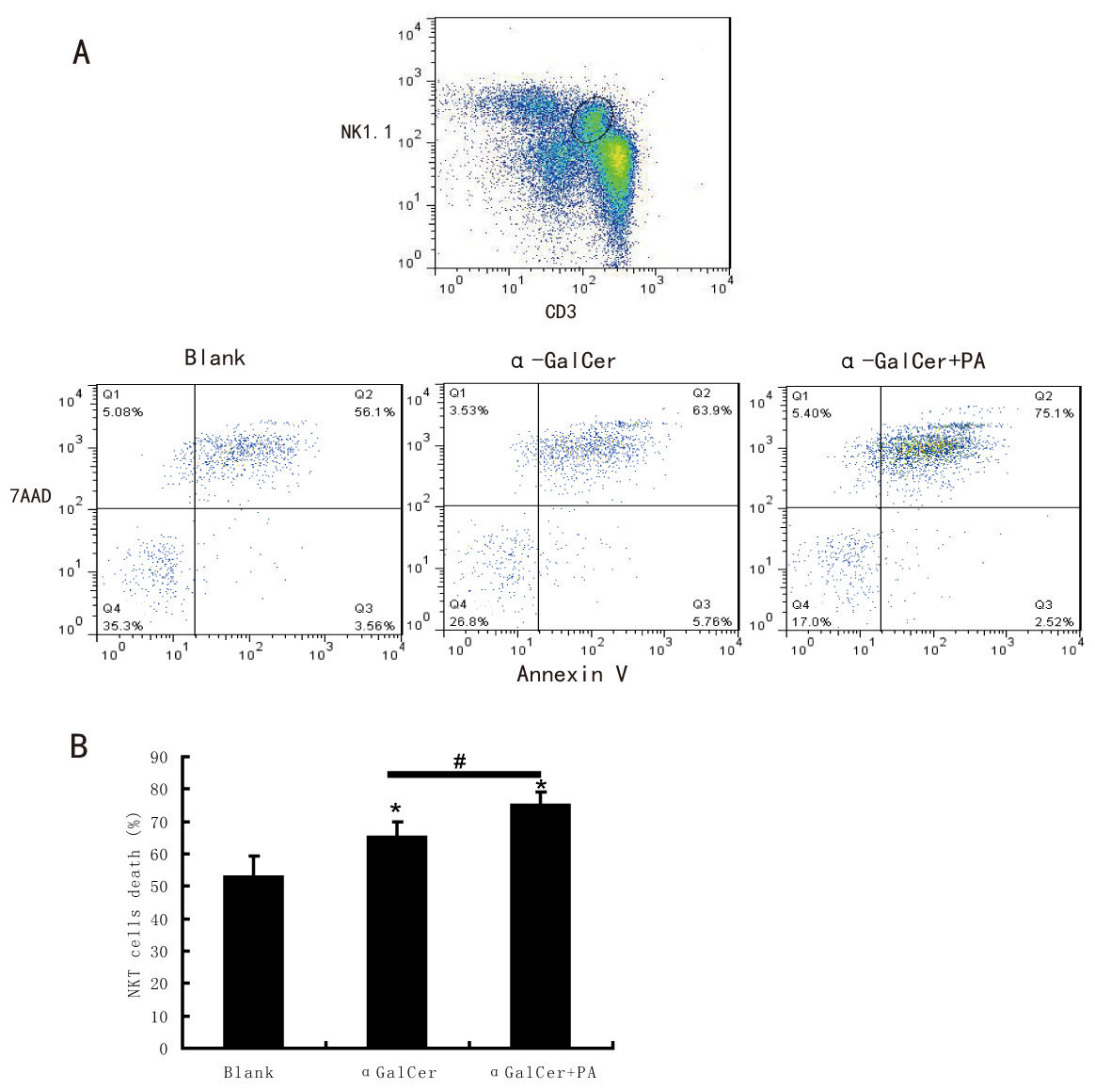
Pro-inflammatory activated Kupffer cells by lipids lead to NKT cells activation- induced cell death. Kupffer cells and HMNCs co-culture system was established as above. After co-culture for 20h, cells were collected and stained with fluorescent antibodies and determined by flow cytometry. Necrosis and apoptosis of NKT cells (gate from CD3+NK1.1+ cells) was defined as Annexin V+7AAD+ cells and Annexin V+7AAD- cells respectively. (A) Representative dot plots of NKT cells (NK1.1+CD3+) necrosis and apoptosis, (B) Mean (±SEM) results of percentage of NKT cell death (apoptosis plus necrosis). *P<0.05 versus Blank, ^#^ *P*<0.05 αGalCer group versus αGalCer+PA group, n=3 experiment.

Our and others previous studies have shown reduced numbers of hepatic NKT cells in the development of hepatosteatosis. However, in our current study, we found Kupffer cells enhanced activation of NKT cells after fatty acids treatment. In order to further study the impact of pro-inflammatory activated Kupffer cell on NKT cells function, we detected the NKT cells apoptosis and necrosis using the same co-culture system as above. As shown in Figure 8A,B, NKT cells (gated in CD3+NK1.1+ cells) co-cultured with Kupffer cells, which first treated with fatty acids and loaded with αGalCer, displayed much more necrosis (Annexin V+ 7-AAD+ cells) and apoptosis (Annexin V+ 7-AAD- cells) when compared to those without fatty acids treatment. We speculated that pro-inflammatory activated Kupffer cells after lipid treatment enhanced NKT cells over activation and induced subsequent cell death, which may contribute to the NKT cells deficiency in the development of NAFLD.

## Discussion

The purpose of the current study was to address effect of lipid treatment on abundance and function of Kupffer cells and further to investigate the potential impact of lipid accumulation on ability of Kupffer cell lipid antigen presentation to activate NKT cells. In the present study, we found high-fat diet significantly increased Kupffer cells numbers and decreased hepatic NKT cells. In addition, high-fat diet or fatty acids treatment induced high levels of pro-inflammatory cytokine expression and TLR4 expression in Kupffer cells. Furthermore, activated Kupffer cells by lipid treatment induced over activation of NKT cells and subsequently led to activation-induced NKT cell death. 

Kupffer cells are important innate immunity components and activation of Kupffer cells induced by different stimuli play the key role in various acute and chronic liver disease[29,30]. Recently, activation of Kupffer cells have been shown an essential element in the pathogenesis of NAFLD similar to other types of liver injury[31]. Selective depletion of Kupffer cells by administration of GdCl_3_ effectively blunted all histological evidence of steatohepatitis and improved insulin resistance in experiment NAFLD[12]. However, the number and function change of Kupffer cells in NAFLD still be controversial. In the current study, we showed significantly increase of Kupffer cell numbers and morporlogical enlargement of Kupffer cells in steatosis liver. In addition, Kupffer cells from high-fat diet mice or treated with certain fatty aicds in vitro showed significant increase in expression of pro-inflammatory cytokine TNF-α and IFN-γ. Findings from the current study suggested activation of Kupffer cells. Actually, we found increased TLR4 gene expression after lipid treatment in vivo and in vitro, which had a central role in macrophages activation. Our results were similar to reports from Leroux A, who has shown that fat-laden Kupffer cells had inflammatory status [32]. However, the mechanism of non-alcoholic steatosis on Kupffer cell activation is not well understood. Lipotoxicity or altered lipid homeostasis is a core event in the development of NAFLD and free fatty acids levels in both peripheral circulation and liver tissues usually increased. Excessive exposure of Kupffer cells to free fatty acids may modulate pathways of inflammation and insulin resistance. Indeed, recent studies have found that saturated fatty acids can directly activate the macrophages by increasing the expression of TLR4 through both Myd88-dependent and TRIF-dependent pathways[25,26]. In addition, Kupffer cells reside within the sinusoidal vascular space and come in contact with a variety of molecular substances such as LPS carried by portal circulation. It is now well established that increased LPS levels due to increased permeability of the gut to LPS are involved in the pro-inflammatory activation of Kupffer cells in NAFLD and NASH[28,33]. Consequently, elevated free fatty acids levels combined with low-grade LPS levels may activate Kupffer cells in the development of NAFLD. 

In the current study, we also detected increased levels of anti-inflammatory cytokine IL-10 gene expression in Kupffer cells, although the extent was lower than that of pro-inflammatory cytokines TNF-α and INF-γ. The seeming opposite results indicated that Kupffer cells had a mixed pro-inflammatory/anti-inflmmatory phenotype but favored pro-inflammatory phenotype polarization under high-fat environment or overnutrition. The alternative explanation may be due to Kupffer cells heterogeneity. Indeed, others have reported some regulatory macrophage subsets expressed high levels of IL-10[34]. 

Lipid homeostasis is disturbed and hepatocellular accumulation of lipids is a key morphologic feature of NAFLD. Previously, we reported that hepatosteatosis induced by high-fat diet was associated with a substantial reduction in resident hepatic NKT cell numbers, and this reduction was comfirmed in different models of obesity[17-19]. Our current findings confirm the previous reports but are inconsistent with two recent reports which failed to observe NKT cell deficiency in mice fed high fat diet (HFD) or high fat and high cholesterol diet (HFCD) [35,36]. The reasons for such discrepancies are unclear, but possible contributing factors include differences in diet composition, feeding duration, and environment in different animal facilities. In addition, NKT cells became activated by dietary lipid with the down-regulation of the NK1.1 expression on them. Indeed, we have observed alteration of NKT cell cytokine profile in HFD mice, and the remaining hepatic NKT cells display pro-inflammatory activation [20]. Furthermore, we found that saturated fatty acids altered hepatocytes endogenous antigen presentation to hepatic NKT cells and contributed to NKT cell depletion[20]. Other authors have showed that endoplasmic reticulum stress and lipid accumulation in hepatocytes of leptin-deficient *ob/ob* mice decrease hepatocytes CD1d expression and led to NKT cell depletion[37]. These studies clearly showed the contribution of hepatocytes to the mechanism of high-fat diet or leptin-deficient induced heaptic NKT cell depletion. However, dietary lipids also cause a significant alteration of NKT cell functional status [20]. This led us to speculate other APCs who possessed immunomodulatory function may be involved in such procession. Kupffer cells are potential APCs and participate in the liver T cell activation and tolerance. In the present study, we found Kupffer cells expressed high levels of CD1d on cell surface and only presented exogenous lipid antigen to activate NKT cells. Interestingly, they showed stronger ability to stimulate NKT cells than that of hepatocytes in the presence of exogenous antigen. Recently, Kremer et al have reported that Kupffer cell-derived IL-12 played key role in depletion of hepatic NKT cells in hepatosteatosis[38]. More recently, it has been shown that fat-laden Kupffer cells had increased ceramide levels, which are involved in member fluidity and phagocytosis[32]. Moreover, some ceramide metabolites can activate NKT cells[13]. However, these results implicated, rather than demonstrated, a role for Kupffer cells in NKT cells activation and depletion. In the current study, using co-culture system, we demonstrated that pro-inflammatory activated Kupffer cells by lipid directly enhanced ability to activate NKT cells and led to NKT cell activation-induced cell death. Recently, Lee et al have demonstrated that Kupffer cell and NKT cell can formed stable contacts in the hepatic sinusoids via the antigen-presenting molecule CD1d in the presence of exogenous lipid antigen, which led to NKT cell activation[39]. Therefore, lipid-derived antigen, such as ceramide, may enhanced Kupffer cell-mediated NKT cells over activation through close cell-cell contact.

However, lipid treatment had no effect on the cell surface CD1d expression in Kupffer cells. We speculated over-activation of NKT cells induced by Kupffer cells may not only through CD1d-mediated lipid antigen presentation but also other signals. Indeed, Kupffer cell-derived cytokines, such as TNFα, play a key role in regulating the phenotype and function of neighbouring parenchymal and non-parenchymal cells[9]. In the present study, Kupffer cells showed the pro-inflammatory profile as TNF-α and INF-γ expression significantly increased after lipid treatment. Furthermore, LPS had synthetic effect in such procession. Our previous studies have demonstrated probiotic improved high fat diet-induced hepatic steatosis and insulin resistance by increasing hepatic NKT cell[40]. The result indirectly indicated that gut-derived bacterial or LPS led to activation of Kupffer cells, which further led to activation and depletion of NKT cells. Therefore, it is possible that a combination of proinflammatory activation of Kupffer cells and cell-cell close contact between Kupffer cells and NKT cells enhanced NKT cells over activation and cell death, subsequently contributing to hepatic NKT cell depletion. Recently, other studies have shown that the liver becomes relatively enriched with NKT cells in rodent and human livers during more severe NASH[41,42]. The apparently opposite outcomes may due to the different microenvironment in different stages of NAFLD. Factors, such as increased Hedgehog (Hh) pathway activity and hepatic expression of IL-15 and Cd1d, that promote NKT cells recruitment, retention, and viability, are induced in rodent and human livers during NASH progression. Thus, additional studies are needed to further investigate the crosstalk between NKT cells and Kupffer cells/macrophages in progressive NAFLD. 

In conclusion, findings from the current study showed lipid treatment induced significant increase of Kupffer cells and pro-inflammatory activation of Kupffer cells. Moreover, pro-inflammatory activated Kupffer cells enhanced ability to activate NKT cells and led to activation-induced cell death, which further lead to hepatic NKT cells depletion in the development of NAFLD. The current study provides new and important information regarding the interaction between Kupffer cells and NKT cells in the development of hepatosteatosis. Further studies based on modulating the phenotype and function of Kupffer cells will be needed to identify the targets for therapy in metabolic-related alternation of the innate immune system and low-grade inflammation.
